# The Discovery of Anæsthesia

**Published:** 1877-07

**Authors:** J. Marion Sims

**Affiliations:** New York.


### ARTICLE III.

### *The Discovery of Anæsthesia.*—Continued.

BY J. MARION SIMS, M. D., NEW YORK.

On the evening of the day (September 30, 1846,) that Morton had this interview with Jackson, he gave the ether to a patient, and extracted a tooth without pain; and on October 16 he gave it in the Massachusetts General Hospital to a patient who had a tumor exsected from the neck by

Dr. John C. Warren. On the next day (October 17,) he gave it to another patient for Dr. Hayward, who exsected a tumor from the arm. He gave it also for Dr. Bigelow with equal success; and from that time it came rapidly into use by the whole profession throughout the civilized world. On October 27, 1846, Jackson and Morton published to the world, by letters patent, the discovery of *letheon* as an anæsthetic, but it was seen at once that their letheon was nothing more or less than pure sulphuric ether. Jackson soon resigned his interest in the patent to Morton, and sent a communication to the French Institute claiming the honor for himself of the discovery of anæsthesia by ether. Morton then set up his claim as the real discoverer, giving Jackson credit only for some unimportant suggestions. While Jackson and Morton were sending bulletins to the Institute of France, Wells sailed for Europe in December, 1846, to lay his claims before the French Institute as the real discoverer of anæsthesia. His mission was a failure, and he returned home in March, 1847, to prepare the documents upon which his claim was to be presented to the Institute. And thus this tripartite war was waged with great fury; Morton and Jackson denying everything to Wells, and denying everything to each other. They denied that nitrous oxide gas had any anæsthetic properties. Wells brought forward his Hartford experience, and he gave the gas for surgeons in general practice, proving that prolonged operations could be performed under its influence. Dr. Marcy exsected a large gland, the patient being under the gas for fifteen minutes; Dr. Ellsworth amputated a thigh; and Dr. Berresford exsected a large tumor under its influence—all in Hartford. But notwithstanding all this, Wells saw nitrous oxide gas supplanted by sulphuric ether as an anæsthetic—ether which he had tried and rejected. He saw his claims as the great discoverer of anæsthesia unrecognized abroad, disputed and set aside at home, and he was disappointed and dispirited. He then went to New York to lay his claims as the discoverer of anæsthesia before the profession of the great metropolis. Soon after his arrival in New York, he showed signs of mental aberration, and on January 14, 1848, in a fit of madness, he ended his life with his own hands.

A few years after the death of Wells, Morton applied to Congress for a grant of money for the discovery of anæsthesia (1853.) The friends of Wells opposed the grant on the ground that Wells was the real discoverer. Then it was that the friends of Long came to the front and opposed the claims of Morton, on the ground that Long was the first discoverer of anæsthesia. The Mortonites admitted that Long was the first to use ether as an anæsthetic, and the first to perform operations under its influence, but they urged that Long's claims were invalid, because he had not published his discovery in some scientific paper. They admitted that Wells tried to make the discovery, but asserted that he failed, because nitrous oxide gas could not produce insensibility to pain. They even attempted to prove this before a Congressional committee appointed for this purpose. Morton declared that nitrous oxide gas never had, and never could, produce the effect claimed by Wells. To disprove this assertion, Prof. John Frederick May, of Washington, went before the Congressional committee and demonstrated the fact that nitrous oxide gas, given according to Wells' plan, could and did produce insensibility to pain.

If nitrous oxide gas can produce insensibility to pain, as Wells claimed, then Wells demonstrated the fact that anæsthesia can be produced by the inhalation of this gas. Let us see how curiously, how providentially, this question has been settled, and settled to the satisfaction of all unprejudiced minds. Colton seems to have been incidentally an important agent in establishing the truth. We have seen how Wells' discovery grew out of Colton's lecture in Hartford in December, 1844. Colton continued his popular lectures on this subject for many years after this. In 1862, he lectured in the town of New Britain, Conn., and, as usual, related how the great discovery of anæsthesia by the use of nitrous oxide gas was made, giving Wells the honor. An old lady present wished to have some teeth extracted; she was afraid to take ether or chloroform, and she requested her dentist, Dr. Dunham, to get Colton to give her the gas for their extraction. He did so, and taught Dr. Dunham how to make the gas. One year after this (1863), Colton returned to New Britain on his usual annual lecture tour, and he found Dunham extensively engaged in extracting teeth under the influence of the gas. Colton then seeing that the extraction of teeth under the influence of the nitrous oxide gas could be made a painless and paying business, induced Dunham to go with him to New Haven, with the understanding that Colton was to lecture and give the gas, and Dunham to extract teeth. After the first day Dunham returned home, and Dr. Smith, of New Haven, took his place, and in a few weeks people came by hundreds to take the gas and get teeth extracted. This experiment convinced Colton that it could be made a great business in a larger field, and he went to New York and opened the Colton Dental Institute, where, since 1863, he and his agents have given the gas to 97,000 persons without an accident.

All this disproves the assertion made by Morton and his adherents. If nitrous oxide gas produces anæsthesia to-day in the hands of Colton and others, it did it in the hands of Wells in 1844, and Wells therefore preceded Morton in the discovery of anæsthesia. Nitrous oxide gas has been used in general surgery by many eminent surgeons in New York, Philadelphia, Baltimore and elsewhere. It has been used successfully in New York, by James R. Wood, Carnochan and others. The writer has used it in difficult and prolonged operations (ovariotomy), requiring thirty, forty, fifty-seven and sixty minutes, and in one case one hour and fifty minutes, and always with the most satisfactory results. And this goes to prove that Wells was right in claiming precedence over Morton in the discovery of anæsthesia by nitrous oxide gas in 1844.

Now let us summarize the facts set forth in the foregoing historic sketch. We know,

1st. That since 1800, the inhalation of nitrous oxide gas produced a peculiar intoxication, and even allayed headache and other minor pains.

2d. That Sir Humphrey Davy proposed it as an anæsthetic in surgical operations.

3d. That for more than fifty years the inhalation of sulphuric ether has been practiced by the students in our New England Colleges as an excitant, and that its exhilarating properties are similar to those of nitrous oxide gas.

4th. That the inhalation of sulphuric ether as an excitant, was common in some parts of Georgia forty-five years ago, though not practised in the colleges.

5th. That Wilhite was the first man to produce profound anæsthesia, which was done accidentally with sulphuric ether in 1839.

6th. That Long was the first man to intentionally produce anæsthesia for surgical operations, and that this was done with sulphuric ether in 1842.

7th. That Long did not by accident hit upon it, but that he reasoned it out in a philosophic and logical manner.

8th. That Wells, without any knowledge of Long's labors, demonstrated in the same philosophic way, the great principle of anæsthesia by the use of nitrous oxide gas (1844.)

9th. That Morton intended to follow Wells in using the gas as an anæsthetic in dentistry, and for this purpose asked Wells to show him how to make the gas, (1846.)

10th. That Wells referred Morton to Jackson for this purpose, as Jackson was known to be a scientific man and an able chemist.

11th. That Morton called on Jackson for information on the subject, and that Jackson told Morton to use sulphuric ether instead of nitrous oxide gas, as it was known to possess the same properties, was as safe, and easier to get.

12th. That Morton, acting upon Jackson's off-hand suggestion, used the ether successfully in the extraction of teeth, (1846.)

13th. That Warren and Hayward and Bigelow performed important surgical operations in the Massachusetts General Hospital (October, 1846,) on patients etherized by Morton, and that this introduced and popularized the practice throughout the world.

In Boston, Mass., a monument has been erected to the discoverer of anæsthesia, but no man is designated thereon by name. The citizens of Hartford, Conn., have erected a bronze statue of Wells (by Bartlett) in their Capitol Park, claiming for him the discovery of anæsthesia. This is as it should be. We have no objection to it; and would suggest that the names of Long, Wells, Morton and Jackson be inscribed on the Boston column, one on each side, as co-discoverers of anæsthesia. The State of Georgia will, at no distant day, erect at its Capitol or its University, a statue of Long, who was unquestionably the first discoverer of anæsthesia.

All the claimants of the honor of discovering anæsthesia are Americans. To each is due a certain measure of credit, but no one man can claim this great honor exclusively. The names of Long, Wells, Morton and Jackson will doubtless be associated as co-laborers in the great work, and to these must be added the immortal name of Sir James Y. Simpson, who introduced chloroform and enlarged the domain of anæsthesia.

Sir James received the highest honor from his government in recognition of the great service he had rendered humanity. I wish we could say the same of our benefactors and government. Our great republic leaves our discoverers and scientists to rest in obscurity and to starve.

Long lost his all during our great civil war, and in his old age he is now being worked to death for the daily bread necessary to support himself and family.

The fate of Wells, Morton and Jackson is most pitiable.

Wells, disappointed in carrying off the honor of the great discovery of anæsthesia, became insane and committed suicide in New York in 1848.

Morton disappointed at not receiving a pecuniary recognition from Congress for his labors, fretted himself into a congestion of the brain. In July, 1868, he returned to New York from Washington in the wildest state of excitement. Fatigue, anxiety and sleepless nights had exhausted his vital powers. Dr. Lewis A. Sayre and Dr. Yale were called to him on the 15th July. They considered his condition as critical, placed him in the hands of a trained nurse, ordered leeches to his temples, cups on the spine, and ice to his head. Dr. Morton would not submit to treatment. As soon as Dr. Sayre left, he ordered his buggy to go to the Riverside hotel, saying he knew he would soon be well if he could get out of the hot city. He drove furiously up Broadway, and through the Central Park. At the upper end of the Park, he leaped from his buggy, and ran to a lake near by to cool his burning brain. Being persuaded to get into his buggy again, he drove a short distance, then leaped out, and jumping over a fence, he fell down in a state of insensibility. He was then taken moribund to St. Luke's Hospital, where he died an hour or two later.

Jackson has been for some time in an insane asylum, hopelessly incurable.

How mournful the fate of these remarkable men! How sad to think that their lives were embittered with envy, jealousy and uncharitableness towards each other! Let us forget their faults, and remember only the good that has resulted from their labors.

It is said that "The evil that men do lives after them." But here the good that these men did will live after them, and live forever.

Vaccination is perhaps the greatest boon ever conferred by science on humanity. Anæsthesia is the next. England gave us the one. America the other. England recognized the labors of Jenner, not, however, in a manner commensurate with the magnitude of his work. America should recognize the labors of Long, Wells, Morton, and Jackson, if not in a manner commensurate with the value of their work, at least to such an extent as to relieve the necessities of their several families, thereby proving that Republics are not always ungrateful. Government aid, voluntarily tendered at this time, would be acceptable to all of them, for they are all really in need of it. Each of these families ought to receive at least one hundred thousand dollars.

I propose, then, that the whole medical profession, North, South, East and West, unite in asking Congress, at its next session, to appropriate this sum, as an anæsthesia fund, to be divided equally between the families of Long, Wells, Morton, and Jackson.

One hundred thousand dollars is a small sum to offer where men have sacrificed their lives for the good of the whole civilized world, leaving their families in straightened circumstances. How small this pittance when measured by the benefits these men conferred on the world!

Let us, as Americans, rise above all party, all prejudice, all sectionalism, and demand of the Government this appropriation for the great work accomplished by these martyrs to science and humanity.—*Virginia Med. Monthly.*

---

